# Digital Shifts in Mental Health Care: A Qualitative Study Examining Staff Perspectives on Integrating Apps Into Their Practice

**DOI:** 10.1192/bjo.2026.11454

**Published:** 2026-06-30

**Authors:** Pinar Ozmen Akkurt, Isabel Mark, Yusuf Saleem, Henry Cheung, Louis Ovonlen

**Affiliations:** 1 South West London and St George's Mental Health Trust, London, United Kingdom; 2 City St George's University of London, London, United Kingdom

## Abstract

**Aims:**

This project examines how mental health professionals perceive and engage with digital phone applications (apps) in clinical practice. ‘MindMeds’, a patient-developed mental health app at Southwest London and St George’s Mental Health (SWLSTG) Trust, was used as a case example. Findings will inform strategies to improve adoption of the MindMeds app, as well as embedding digital tools into clinical practice, workflow embedding and aligning with Trust digital health priorities.

**Methods:**

A qualitative staff survey was distributed via Microsoft Office Forms to all SWLSTG Trust staff. Recruitment was conducted through mass email and Trust communications. The survey collected key demographic information and included Likert-scale items assessing digital confidence and familiarity with mental health apps. Semi-structured free-text questions explored staff awareness, attitudes towards digital apps, key enablers and barriers, clinical considerations of how apps fit into workflows, as well as wider systemic factors such as the Trust’s position on digital innovation.

Qualitative data were analysed using reflexive thematic analysis following Braun and Clarke’s six-step framework, with quantitative findings used to contextualise findings and gauge the prevalence of emerging themes.

**Results:**

31 responses were received and thematic saturation was achieved. Although reported digital confidence was high (median 8/10), awareness and regular use of digital apps (particularly MindMeds) was low. 45% of respondents had never heard of the app despite multiple publicity efforts.

Five key themes emerged: 1) Time and workload pressures; 2) Concerns regarding safe integration with electronic patient records; 3) Uncertainty regarding evidence base and regulation; 4) Limited awareness and visibility of Trust-endorsed apps; 5) Variable patient digital literacy and access.

Challenges were particularly pronounced in inpatient, crisis, and CAMHS settings. Clinicians consistently reflected that apps were more likely to be adopted when aligned with existingworkflows, had a clear clinical purpose, and allowed patient-generated data to be accessed within the electronic patient record.

**Conclusion:**

Digital mental health apps remain poorly embedded in routine practice in SWLSTG Trust, with MindMeds gaining little traction despite extensive development. Uptake appears to be limited less by staff attitudes, and more by structural, governance, and workflow barriers. Improving adoption may require simplification of digital tools, integration with core clinical systems, and clear Trust-level governance, training, and operational support. Focusing on high-value use cases alongside co-design and targeted awareness efforts may enable more effective and equitable integration of digital apps into mental health care.

